# Age, Body Mass Index (BMI) And Cognitive Difficulties In Appalachian West Virginia

**DOI:** 10.1093/geroni/igab046.3560

**Published:** 2021-12-17

**Authors:** Camila Romero, Moyosoreoluwa Jacobs, Bernard Schreurs

**Affiliations:** 1 WVU, West Virginia University, West Virginia, United States; 2 West Virginia University, Morgantown, West Virginia, United States; 3 WVU, Rockefeller Neuroscience Institute, WVU, West Virginia, United States

## Abstract

Several known factors exacerbate the risk of cognitive difficulties among older adults. In addition to place-based disparities, body weight also predicts cognitive health. In fact, overweight and Obese BMI (Benito-Leon et al., 2013) and underweight BMI (Xiang & An, 2015) are risk factors for cognitive difficulties. Whether the effect of BMI operates similarly across age among adults facing place-based disparities is not clear. In order to better understand the role of BMI among adults already at-risk for health disparities, we used the 2018 BRFSS data to examine the relations among these variables among the 4817 West Virginian adults in the BRFSS database. Approximately 16.5% had difficulty making decisions or remembering. Approximately 73% were overweight or obese, 26% were healthy BMI, and 1% were underweight. A multinomial logistic regression was conducted to examine cognitive difficulties. Healthy BMI and ages 25 – 44 years were the reference groups. The omnibus test was significant, χ2 (5, N = 4817) = 38.71, p < 0.0001. Age and BMI uniquely contributed to the classification. Post hoc inspection of the Odds Ratios showed that adults ages 60 years and older with obesity were 1.27 times more likely to report cognitive difficulties, while those who were underweight were 3.74 times more likely to report cognitive difficulties. That individuals over age 60 with an obese or underweight BMI report more cognitive difficulties highlights the intersection among age, obesity and location on cognitive health disparities in West Virginia.

